# Contact prediction in protein modeling: Scoring, folding and refinement of coarse-grained models

**DOI:** 10.1186/1472-6807-8-36

**Published:** 2008-08-11

**Authors:** Dorota Latek, Andrzej Kolinski

**Affiliations:** 1 Faculty of Chemistry, University of Warsaw, Pasteura 1, 02-093 Warsaw, Poland

## Abstract

**Background:**

Several different methods for contact prediction succeeded within the Sixth Critical Assessment of Techniques for Protein Structure Prediction (CASP6). The most relevant were non-local contact predictions for targets from the most difficult categories: fold recognition-analogy and new fold. Such contacts could provide valuable structural information in case a template structure cannot be found in the PDB.

**Results:**

We described comprehensive tests of the effectiveness of contact data in various aspects of de novo modeling with CABS, an algorithm which was used successfully in CASP6 by the Kolinski-Bujnicki group. We used the predicted contacts in a simple scoring function for the post-simulation ranking of protein models and as a soft bias in the folding simulations and in the fold-refinement procedure. The latter approach turned out to be the most successful. The CABS force field used in the Replica Exchange Monte Carlo simulations cooperated with the true contacts and discriminated the false ones, which resulted in an improvement of the majority of Kolinski-Bujnicki's protein models. In the modeling we tested different sets of predicted contact data submitted to the CASP6 server. According to our results, the best performing were the contacts with the accuracy balanced with the coverage, obtained either from the best two predictors only or by a consensus from as many predictors as possible.

**Conclusion:**

Our tests have shown that theoretically predicted contacts can be very beneficial for protein structure prediction. Depending on the protein modeling method, a contact data set applied should be prepared with differently balanced coverage and accuracy of predicted contacts. Namely, high coverage of contact data is important for the model ranking and high accuracy for the folding simulations.

## Background

Information on a pattern of long-range interactions, even very sparse, can be crucial in protein structure prediction[1,2]. Long-range interactions determine a protein structure mainly by placing the building blocks (helices or beta strands) at the appropriate distance between their residues.

Whereas short-range information, such as the type of secondary structure, can be predicted in most cases with high accuracy (70–80%)[3] on the basis of a protein sequence, long-range contact predictions are still of rather low accuracy (at most 20%, according to the CASP6 results[4]). Such low accuracy of contact predictions, although well above random (by a factor of more than 11)[5], is not enough for the direct reconstruction of the 3D protein structure using for example distance geometry methods, common in NMR structure determination [6-8]. It is mainly due to the large number of false contact predictions[4]. Nevertheless, if we use this kind of data only as additional and complementary information in protein folding simulations, there is a good possibility that the force field will cooperate with the true contacts and discriminate against the false contacts, biasing the simulations towards the native-like structures. The main aim of this work was to establish to what extent this hypothesis is true.

A frequently used definition of a term "contact" states that two residues are in contact when the distance between their Cβ atoms is smaller than 8 Å[4,9-11]. There are other, and perhaps more precise, definitions[12]. However, due to the conventions employed by most of the contact-prediction servers, we adhere to the Cβ-based definition. A term called "sequence separation" divides contacts into short-range (for which contacting residues are separated by at least 6 and at most 12 residues in a sequence), medium (12–24 residues) and long-range contacts (at least 24 residues)[4]. If we generate a symmetric, two dimensional matrix with rows and columns corresponding to a protein sequence, in which contacts between appropriate residues are binary depicted, we obtain a contact map. Such contact maps can be generated easily from a 3D protein structure, predicted from a protein sequence alone or obtained experimentally from NOESY spectra[13].

Early methods for contact map prediction were based on the observation that residues which are closed in space often mutate in tandem. This was called correlated mutations (CMA) and was detected by the analysis of multiple sequence alignments[9]. The addition of other types of data, such as sequence conservation, predicted secondary structure and solvent accessibility, was necessary to improve rather weak performance of contact predictions based on CMA only[14,15]. The major breakthrough in contact prediction resulted from the application of machine learning methods such as neural networks[11,14,16]. Current methods also employ hidden Markov models often combined with threading[4,17], support vector machines [5,18] and genetic programming[19]. Detailed information about the selected contact prediction methods is shown in Table 1.

**Table 1:** Description of the selected nine CASP6 contact predictors.

Contact predictors	Method	Input data	Accuracy [%]	Coverage [%]
Baker	Neural network	Contact predictions from 24 servers, predicted by JUFO secondary structure, amino acid properties, PSI-BLAST generated PSSM matrix, length of a protein sequence	25.5	3.7
PROFcon	Feed-forward neural network with back-propagation	evolutionary profiles obtained using PSI-BLAST, predicted secondary structure and solvent accessibility, sequence conservation, biophysical features and "complexity" of residues	24.2	3.6
Baldi-group-server	RNN – Recursive neural network	PSI-BLAST generated sequence profiles, correlated mutations, predicted secondary structure, solvent accessibility	21.9	2.9
GPCPRED	Genetic programming with self-organizing maps	PSI-BLAST generated sequence profiles, sequence separation	17.4	2.7
Karypis	Support Vector Machines	Sequence profiles, correlated mutations from multiple sequence alignment analysis, sequence conservation, sequence separation, predicted secondary structure	11.0	1.5
KIAS	CMA analysis	Multiple sequence alignment, hydrophobic packing of residues (data obtained from sequence conservation and hydrophobicity)	11.0	1.7
SAM-T04	Neural network	Alignments, predicted secondary structure and propensities of residues in contact	9.6	1.43
Hamilton-Huber-Torda	Feed-forward neural network	Mutational correlations from multiple sequence alignments, biophysical class of contacting pair of residues, predicted secondary structure, sequence separation, length of protein sequence	9.1	1.3
CORNET	Neural network	PSI-BLAST generated sequence profiles, correlated mutations and sequence conservation, sequence separation	2.5	0.34

Mean accuracy and coverage was evaluated for all NF and FR-analogy targets. Here, we defined the accuracy and the coverage in the same fashion as in[4] for top N/5 predicted contacts with a sequence separation of 12: $\begin{array}{cc} Acc=\frac{N_{true\;positive}}{N_{true\;positive}+N_{false\;positive}}\cdot 100\% & Cov=\frac{N_{true\;positive}}{N_{native}}\cdot 100\% \end{array}$.

The contact prediction approach is usually not optimized to find the closest homologues of a given protein[4], unlike in the case of comparative modeling or fold recognition methods [20,21]. Consequently, it could be more sensitive in detecting more distant structure similarities with respect to traditional fold recognition or comparative modeling approaches[4,14]. This seems to be especially useful in the structure modeling of new folds, for which meta-predictors (Bioinfo[22] etc.) based on the consensus of fold recognition methods do not yield any reliable templates. Therefore, our work focuses on the contact-based structure prediction of targets from the two categories defined in CASP6[23]: New Fold (NF) and Fold Recognition – Analogy (FR/A), for which producing a reliable template structure was extremely difficult or impossible.

Despite the significant potential of contact prediction, typical de novo protein structure prediction methods still do not use this kind of data regularly in modeling pipelines, except for a few examples[4,24-29] Probably the main reason is the difficulty in implementation of such low accuracy data in the reliable protein structure prediction schemes. As we mentioned before, contact maps predicted on the basis of protein sequences are not accurate enough for a direct and thus very fast and simple reconstruction of 3D protein structures. The CABS algorithm, which is used in this work, enables incorporation of different kinds of structural data, in the form of distance or angular restraints, not necessary accurate, such as for example chemical shifts[30]. This feature of CABS encouraged us to test the low accuracy contact data at various stages of protein structure modeling.

We focused our test on CASP6 predictions because many contact predictors, after moderate success in the CASP6, abandoned the development of their methods, probably because of the limited usage of such data by structure predictors, which resulted in slightly worse performance in CASP7[31], in which the targets were apparently more difficult (many with good templates difficult to detect) as well. Moreover, the Kolinski-Bujnicki group did not take part in CASP7 and thus reproducing the same conditions, as those typically applied in CASP competitions, could not be possible. The aim of this work is to propose a novel, predicted-contact-assisted approach to protein structure prediction which could be used in high-throughput modeling pipelines which are especially inevitable in CASP – like, large-scale experiments.

An important issue in de novo modeling, enhanced by contact prediction, is to establish a minimal level of accuracy and coverage of contact data that could be still useful for structure prediction. Typically, increasing the coverage to obtain the majority of the important contacts decreases the average accuracy of the whole prediction. This interplay between coverage and accuracy seems to be crucial for successful protein structure prediction (see **Results and Discussion**). It is important to note that most of the false contact predictions are shifted by one or two residues with respect to the true native contacts and the minority of all predicted contacts are completely wrong predictions (see Table 2 in **Methods**). As it is shown in this work, these moderately shifted contacts can still be useful, provided that they are implemented as very soft biases in the folding algorithms, as in the CABS algorithm[32]. This fact should be taken into consideration while developing contact prediction methods.

**Table 2:** Results of the contact-based ranking of Kolinski-Bujnicki's models for NF and FR/A CASP6 targets.

Set of contact data	Number of predicted contacts	Accuracy δ = 0 [%]	Coverage δ = 0 [%]	Accuracy δ = 2 [%]	False contacts (δ > 5) [%]	ΔRMSD [Å]	Spearman corr. coeff. (GDT-TS)	Spearman corr. coeff. (RMSD)
N/2 top-scoring contacts from each of the best two predictors ^(a)^	N	19.58	12.91	49.37	19.59	1.069	-0.300	0.329
N/2 top-scoring contacts from each of the best three predictors^(b)^	1.5 N	17.21	15.22	46.90	21.13	1.453	-0.321	0.275
Consensus of the whole data from the best three predictors	N/2	23.94	9.12	53.17	17.93	1.393	-0.325	0.344
	N	18.90	14.44	49.17	20.35	1.387	-0.333	0.340
	1.5 N	15.53	17.83	46.60	22.15	1.453	-0.329	0.288
Consensus of the whole data from the best five predictors^(c)^	N/2	23.78	8.97	52.47	17.67	1.272	-0.196	0.360
	N	19.78	15.07	51.01	20.04	1.498	-0.350	0.348
	1.5 N	16.64	18.91	47.70	22.11	1.443	-0.338	0.400
Consensus of the whole data from all nine predictors	N/2	24.98	9.50	51.69	20.56	1.252	-0.238	0.432
	N	20.63	15.74	50.58	21.85	1.365	-0.333	0.400
	1.5 N	18.08	20.37	49.35	23.39	1.322	-0.342	0.440

^(a) ^Predictors: Baker and PROFcon

^(b) ^Predictors: Baker, PROFcon, GPCPRED

^(c) ^Predictors: Baker, PROFcon, GPCPRED, Karypis, SAM-T04

Spearman correlation coefficients together with the average difference of Cα RMSD of the best model and the first ranked model (ΔRMSD) were computed for each set of contact data. The average ΔRMSD of the Kolinski-Bujnicki group was 1.484 and the Spearman correlation coefficient: 0.213 (RMSD) and -0.138 (GDT-score). These values were improved for every set of contact data. Apart from the mean accuracy and the mean coverage of contact data, averaged over all targets, we also present the percentage of semi-accurate contacts (shifted with respect to the real by at most two residues) and totally false contacts (shifted by more than five residues).

De novo modeling could be enhanced by contact prediction in various ways. First of all, contact maps could be used to generate restraints which bias either de novo folding simulations[28] or refinement simulations which start from preliminary models obtained via more straightforward simulations (see **Methods**). In the other approach, predicted contacts can be used as a part of various scoring functions for ranking protein models generated by any de novo or fold recognition method[27,33]. In this work, we verify whether these three approaches could enhance protein structure prediction carried out using the CABS modeling tool[32] and if so, to what extent. Our results are compared to the CASP6 predictions of the Kolinski-Bujnicki group[34] who used the CABS algorithm (de novo method)[32] and the Frankenstein-3D (a fold recognition tool)[35] without any information about predicted contacts.

## Methods

### Preparation of data sets for predicted contacts

In this work we used contact data provided in CASP6 by nine predictors performing best and average: Baker group[4], GPCPRED[19], Hamilton-Huber-Torda group[36], KIAS[37], Karypis group[5], SAM-T04[38], baldi-group-server[39], CORNET[40] and PROFcon[11] (see Table 1). Contacts provided by the average-performing groups were added to our data because in the real CASP experiment the final performance of each group would not be known a priori and it would rather be impossible to choose only the best contact predictions for the structure modeling. We used contact data only for targets from NF (New Fold) and FR/A (Fold Recognition Analogy) categories because in the remaining CM (Comparative Modeling) and FR/H (Fold Recognition Homology) categories template structures could be found relatively easy in the PDB and thus the contact prediction did not provide any additional and valuable information.

Original data sets contain different numbers of predicted contacts (several, N/2, N; where N is sequence length, or even a few thousands) and assume different minimal sequence separations (from 1 to 12). Such heterogeneous data had to be converted into sets of restraints which could be most beneficial for protein structure prediction. For this reason, we tested different data sets (see Table 2) which contain either contacts from all nine predictors or from the selected best-scoring predictions. All the nine contact predictors used different methods and any consensus selection could perhaps cover-up the imperfections of a single method and thus improve the accuracy of the combined data. Such an approach was successfully exploited by Baker et al. in CASP6[4]. We compared the results of the modeling supported by the consensus contact data with the results obtained using the data set of N/2 top-scoring contacts provided by each of two or three best predictors. In the latter approach, instead of processing the preliminary consensus which could filter out the false contacts, we allow the CABS force field to cooperate with the large sets of predicted contacts in order to obtain some kind of consensus during the simulation. Moreover, in this approach, top-scoring contacts, which were predicted by more than one predictor, were placed in a set of restraints more than once. Consequently, the strength of bias generated by these contacts became somehow multiplied.

When generating all tested sets of contact data we chose only those contacts from each predictor for which the contacting residues were separated in the sequence by at least twelve residues. It has been observed in simulations (data not shown) that the short-range contacts with sequence separation below 12 could be obtained using solely the CABS force field due to its well optimized short-rang potentials. Such short-range contacts are typically responsible for the loop formation at the end of a helix or a beta strand. The CABS algorithm is able to reproduce such ending of secondary structure elements itself, provided the predicted secondary structure is of a reasonable accuracy. This was tested in various cases of loop-modeling[32]. Ignoring the predicted contacts with sequence separation below 12 eliminates also their over-expression in the data sets, which is caused by the fact that they are typically much easier to predict than the medium and long-range ones[14]. Those medium and long-range contacts seem to be more important in the structure modeling because they are sometimes difficult to reproduce in template-free CABS simulations.

For the consensus sets of contacts we purposely ignore the fact that some predictors optimised their contact prediction to the specific number of contacts (e.g. N/10[19,36] or N/2[11]). The reason for this was the limitation of their methods which could score some relevant contacts very low. Consequently, a minor improvement in accuracy of the final set of contacts obtained by rejecting the low-scoring contacts could be at the serious expense of coverage.

### Contact-based ranking of CASP6 models

We employed the sets of contact data described in Table 2 in various protein structure modeling procedures. First of all, we used the predicted contacts for ranking five models for each target submitted to the CASP6 server by the Kolinski-Bujnicki group. The purpose of this test was to verify whether the selection of the best models (far from perfect in CASP6) could be improved by scoring these 5 submitted models with predicted contacts, i.e. if the modified scoring function would correlate better with the RMSD of the models than the scoring function based solely on the CABS force field and the results of clustering the simulation trajectories. We tested different kinds of the contact-based component of the scoring function (linear, square root and quadratic dependence of the deviations between the observed and predicted Cβ-Cβ distances). The highest correlation coefficients were observed for the linear scoring function and all the results presented here are obtained using the following form of this function:

(1) $$score(i,j)=\{\begin{array}{cc} 0 & d(i,j)\leq 8\text{Å}\\ f\cdot d(i,j) & d(i,j)>8\text{Å} \end{array}$$

where: f is a scaling factor (here equal to 1.0), d(i,j) is the difference between the observed Cβ-Cβ distance and the reference distance of 8 Å (a standard cut-off distance in the contact definition[4]), i and j are residues predicted to be in contact. The final score for the given structure and the predicted contact set is computed as a sum of all i,j pairwise scores.

### REMC simulations of CASP6 targets with contact-based restraints

The usefulness of the predicted contacts in protein modeling was also tested in the structure refinement of the final model and in contact-assisted de novo folding. In both cases we used the CABS modeling tool[32]. The CABS algorithm predicts protein structures on the basis of their sequences. It employs a simplified lattice representation of a protein. Each residue is represented by four centers of interactions: Cα united atom, Cβ atom, a united atom in the side-chain center of mass and a united atom representing the peptide bond, located in the center of a Cα-Cα pseudobond. The conformational space of a model protein is explored by the Replica Exchange Monte Carlo method (REMC), a very efficient technique for finding the global energy minimum [41]. The conformational energy of a protein is evaluated by several knowledge-based potentials which bias the model chain towards protein-like conformations. The force field includes long-range orientation-dependent contact-type potentials, short-range sequence-dependent potentials and a hydrogen bonding potential. The details of the CABS force field design and the description of its applications in generalised comparative modeling, folding pathway prediction, docking and de novo modeling and modeling supported by sparse experimental data can be found in other publications[30,42-44].

Apart from the protein sequence, for a better performance, CABS also requires some information about the likely secondary structure coded in a three letter code (E-beta structure, H-helix, C-coil or loop). Such a secondary structure is typically predicted quite accurately by different servers (e.g. PSIPRED[45]). In order to reproduce the same conditions of the folding simulations as those applied by the Kolinski-Bujnicki group during the CASP6, except for the additional contact data, we used the same predicted secondary structure and the same version of the knowledge-based potentials as employed during CASP6.

The structure refinement supported by the contacts predicted was conducted in several low-temperature REMC simulations with different sets of parameters and different sets of contact data. In every simulation the five protein models submitted to the CASP6 server by the Kolinski-Bujnicki group were used as the initial replicas. The lowest temperature starting replica was the first model submitted in CASP6 by this group. Employing these initial conditions, we refined the structures obtained by the CABS-based de novo modeling combined with evolutionary information from Frankenstein-3D[34].

The predicted contacts were used in the refinement as restraints imposed onto Cβ-Cβ distances in the form of square root potential, tested previously[32]:

(2) $$\begin{array}{ll} E=\sum_{k}f(\sqrt{d_{k}^{current}-d_{k}^{predicted}}-1) & \begin{array}{cc} for & d_{k}^{current}>d_{k}^{predicted}+1 \end{array}\\ E_{k}=0 & \begin{array}{cc} for & d_{k}^{current}<d_{k}^{predicted}+1 \end{array} \end{array}$$

Here, k is a number of a contact between residues i and j and E_k _– is a component of the contact potential associated with this contact; $d_{k}^{current}$-is a current distance between Cβ atoms of residues i and j; $d_{k}^{predicted}$ – is a predicted maximum distance between these atoms, set to 8 Å; f is a scaling factor (typically between the value of 0.1 and 1). The energy component of the contact potential is added to the total conformational energy only when its value exceeds the certain cutoff depending on the expected quality of the predicted contacts. This feature of the CABS force field is very useful when the restraints data is sparse and inaccurate, because it enables to incorporate restraints into the simulation only when the protein conformation seriously disagree with most of them and discarding them when only a small fraction of restraints (typically 20–30%), which could be false anyway, are not satisfied. The same type of restraint potential was used in de novo folding simulations. Contact-assisted de novo folding simulations were started from random polypeptide conformations. The first stage of de novo folding was processed without contact-based restraints, which were implemented only in the second stage of the simulation. The preliminary simulation with the CABS force field only facilities a very efficient search for the local minima of the free-energy in the enormous conformational space, especially in the case of large protein structures, without any restrictions from restraints [30]. Typically, in the preliminary simulation, a random polypeptide chain yields a protein-like secondary structure, but its overall topology is not set.

Preliminary testing and optimisation was processed for the limited number of 14 CASP6 targets (T0198, T0199-3, T0209-1, T0212, T0215, T0230, T0235-2, T0239, T0248-1, T0262-1, T0272-1, T0272-2, T0280-2, T0281) in the series of contacts-supported de novo folding and refinement simulations. After these testing simulations we chose the best performing contact data set on the basis of the RMSD of the obtained models. It was the contact data obtained from the best two predictors (Baker and PROFcon – see **Results and Discussion**, Table 3). We used this contact data set in simulations for all the remaining targets from NF and FR-analogy categories.

**Table 3:** Comparison of different approaches to contact-based modeling tested in this work.

Set of contact data^(a)^	Number of predicted contacts		avg. Cα RMSD [Å]			
		
		Contact-based ranking	De novo folding		Refinement	
			First ^(c)^	Best ^(d)^	First ^(c)^	Best ^(d)^
N/2 top-scoring contacts from each of the best two predictors	N	9.58	8.93	7.53	7.69	7.10
N/2 top-scoring contacts from each of the best three predictors	1.5 N	9.96	8.69	8.02	8.23	7.36
Consensus of the whole data from the best three predictors	N/2	9.82	8.15	7.14	8.28	7.17
	N	9.80	8.82	7.35	8.11	7.30
	1.5 N	9.81	9.21	8.03	8.68	7.73
Consensus of the whole data from the best five predictors	N/2	9.83	8.92	7.92	8.44	7.02
	N	9.91	8.94	7.65	7.89	7.21
	1.5 N	9.79	8.70	7.57	7.71	7.11
Consensus of the whole data from all nine predictors	N/2	9.70	9.26	7.98	8.06	6.44
	N	9.77	8.79	7.52	7.63	6.41
	1.5 N	9.64	8.42	7.07	8.02	6.99

^(a) ^The same as in Table 2.

^(b) ^RMSD computed only for models ranked as first.

^(c) ^RMSD component computed only for that model of each target which was likely to be selected as the first model (the most probable), because it was a centroid structure of the most populated cluster obtained in a simulation.

^(d) ^RMSD component computed only for the best model of each target (a centroid structure of a cluster which was most similar to the native structure).

The performance of each method is presented as the Cα RMSD of the final protein model averaged over 14 targets selected, for the sake of shortening computation time, from NF and FR/A categories (i.e. T0198, T0199-3, T0209-1, T0212, T0215, T0230, T0235-2, T0239, T0248-1, T0262-1, T0272-1, T0272-2, T0280-2, T0281). The average Cα RMSD of the first models for this subset of 14 CASP6 targets for the Kolinski-Bujnicki group was 10.27 and we observed improvement of this value for every method and every contact data set. The lowest average RMSD was obtained in the case of the refinement simulations with contact restraints based on the data from the best two predictors.

In de novo folding and in the refinement simulations the final protein models were selected from the CABS trajectories as centroids of the most populated clusters using the hierarchical clustering procedure, described elsewhere[46]. The rebuilding of all-atom structures from the CABS Cα only traces was done using the BBQ program from the BioShell package[46].

## Results and discussion

### Predicted contact-based ranking of CASP6 models

The first question asked was whether the ranking of the models provided by the Kolinski-Bujnicki group could be improved by the ranking based on the contact-dependent scoring function. Because of the limited size of the data set which was too small to assume normal distribution, we employed the Spearman rank order correlation instead of the commonly used Pearson correlation. Spearman correlation coefficients were evaluated for each target separately and then averaged over all targets to obtain the final correlation coefficient for a given set of contacts. The Spearman rank-order correlation was used for example by Feig et al. in the evaluation of CASP4 protein models obtained by different modeling methods, from comparative modeling to de-novo folding[47].

The contact-based scoring function improved the ranking of models submitted by the Kolinski-Bujnicki group for every set of contacts considered in this work. As it is shown in Table 2, the more diverse (from different predictors) and the larger data set is considered, the higher correlation coefficients are observed. The highest RMSD-based correlation coefficient (0.440) is for the set of 1.5 N consensus contacts derived from all the nine tested predictions. The correlation coefficient between the RMSD-based ranking and the ranking provided in CASP6 by the Kolinski-Bujnicki group was only 0.213, so the contact-based ranking led to a significant improvement of the best model selection.

In the case of protein models which differ significantly from the native structures, the GDT-TS (global distance test) measure is used more often in evaluating the results than the RMSD values[47]. Though RMSD correlates with GDT-TS values, GDT-TS is more sensitive in distinguishing different protein topologies than RMSD[47]. In Table 2, apart from the correlation coefficients computed using the RMSD measure, we also present correlation coefficients between the GDT-TS-based ranking of protein models and the ranking based on the predicted contacts. The GDT-TS scores were obtained using the TMscore program[48]. If a protein model is similar to a native structure, the GDT-TS score is high. Thus, the best correlation would be for the Spearman correlation coefficient equal to -1.

The contact-based ranking of models correlates better with the RMSD than with the GDT-TS. The correlation coefficients based on the GDT-TS ranking were obtained in the range from -0.350 to -0.196 (for the Kolinski-Bujnicki group in CASP6: -0.138). Such results, though obtained using a different testing set, are comparable with those of Feig et al. (correlation coefficients for assessing CASP4 models: from -0.407 to -0.218, depending on the type of a physical energy function used)[47].

Apart from the correlation coefficients, we also present the ΔRMSD value, which is the difference in Cα RMSD between the first ranked model and the best model, averaged over all targets (see Table 2). Such measure of the performance of ranking methods is widely used[49]. In this case, we improved Kolinski-Bujnicki's results in the best case by about 0.4 Å (from 1.484 to 1.069).

Correlation coefficients and average ΔRMSD values were compared in Table 2 with accuracy and coverage of the given contact sets, averaged over all targets. The accuracy of the data was computed considering the δ analysis by Ortiz et al[28]. The value of δ, defined as a maximum shift in the predicted contacts compared to the real ones, was chosen as equal to 0 (accurate contacts), 2 (semi-accurate contacts) and more than 5 (wrong contacts). Generally, the coverage is higher when more contacts are taken into the set but in that case the accuracy decreases. Also, when the coverage improves, the number of completely wrong contacts (shifted by more than 5 residues) increases and the fraction of semi-accurate contacts decreases. The maximum number of shifted residues (δ = 5) which could be still useful in structure modeling is chosen independently of the type of the secondary structure of the target protein. However, it is worth noticing that a 5-residue-long shift in a contact set could be more destructive for extended beta-type structures than for compact helix structures.

Generally, models are better ranked (higher correlation coefficients) when the contact data are of high coverage (e.g. 1.5 N contacts instead of N/2). However, if we examine only the difference between the first ranked and the best model (ΔRMSD), better results are obtained when the accuracy is high, but not the coverage (e.g. sets of N/2 and for N contacts instead of 1.5 N).

**Figure 1:**
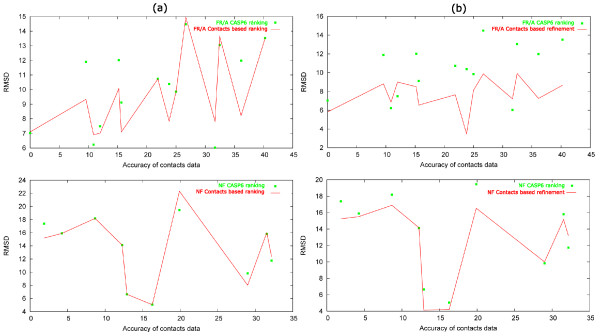
**Comparison of the CASP6 results of the Kolinski-Bujnicki group with post-CASP contact-based modeling**. Results of the contact-based ranking of Kolinski-Bujnicki's models from CASP6 is shown in (a). The scoring function was based on the contact data set from the best two predictors (Baker and PROFcon). The accuracy of the contact data used for scoring 5 models of each target is plotted against the RMSD of the model ranked as first in CASP6 by the Kolinski-Bujnicki group (green squares) and against the RMSD of the protein model ranked as first by the contact-based scoring function (red lines which join corresponding points). Results for NF and FR/A categories are presented separately. The most significant improvement is observed in the case of FR/A targets with the accuracy of the contact prediction range of 15–30%. In a similar way the results of the refinement simulations are presented in the right-hand panels (b). The refinement simulations performed better than the post-simulation ranking of the models.

In most cases (see Figure 1a), the Cα RMSD of the models selected as first according to the contact-based criterion was lower or the same as the RMSD values of the Kolinski-Bujnicki's first models. Only one model out of 14 in the FR-analogy category and two models out of 10 in the NF category were worse than the first models selected by the Kolinski-Bujnicki group. We managed to improve the prediction of five models in the FR category and two models in the NF category. The quality of the remaining models did not differ significantly from the CASP6 results.

The two targets from the NF category for which the contact-based scoring function failed in the selection of the best models were T0241-2 and T0216-2. The reason for such weak results is that all models provided by the Kolinski-Bujnicki group were of a very poor quality (RMSD = 16–24 Å). In such cases, agreement with the predicted contacts may not correlate with the quality of models.

The most significant improvement in the models' ranking was obtained for targets for which the accuracy of contact data was in the medium range of 15–30%. Below this range, the contact-based selection did not improve the quality of models significantly, but, and this is perhaps more important, did not worsen the results. Above this range the improvement in most cases was not significant, probably because such high accuracy of contact data means that contact prediction was straightforward. Such high accuracy of contact prediction was observed only for FR targets, for which some similar protein structures could be detected by a sensitive fold recognition method and was certainly detected by Frankenstein-3D used by the Kolinski-Bujnicki group. Thus, contact prediction in these cases did not provide any additional information.

**Figure 2:**
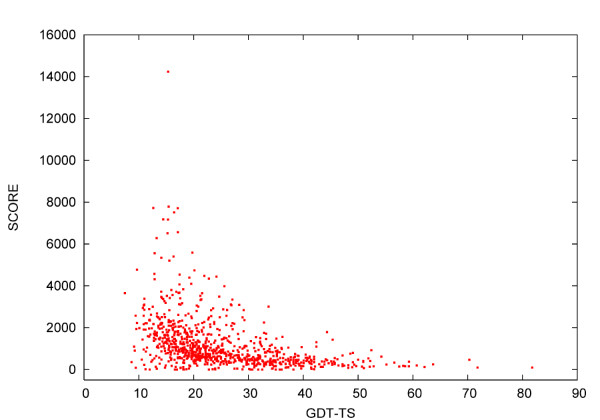
**Contact-based scoring of NF and FR/A models submitted as first by all groups in CASP6**. For each model, the final score computed using the scoring function, which was based on the contacts provided by the Baker group and PROFcon, was plotted as a function of GDT-TS. Although most of wrong or low quality models (with GDT-TS < 40) could be discarded by the contacts based scoring function, it seems inevitable to use some additional discriminating tools for assessing models with GDT-TS > 40.

As the results of the contacts-based scoring of the Kolinski-Bujnicki's models were encouraging, we tested the scoring function for models submitted by other groups in CASP6 (see Figure 2). While, the contact-based scoring function is able to distinguish between completely wrong protein models (GDT-TS < 40) and those nearly native (see Figure 2) it needs to be combined with other kinds of data or scoring functions while accessing the models much closer to the near-to-native structures (with GDT-TS > 40).

### Refinement of CASP6 models with predicted contacts

Predicted contacts are more valuable in refinement simulations using CABS than in the post-simulation ranking of the most probable models (see Figure 1). It is a consequence of the fact that, in contrast (for instance) to Rosetta[50], CABS was developed not to generate many distinct protein conformations and rank them after the simulation, but rather to bias the protein conformation towards its single global free energy minimum. The improvement of protein models after the CABS refinement was significant in the case of targets from the FR/A category (11 targets out of 14; even by about 6 Å). In the case of NF targets we managed to decrease the RMSD value of 7 out of 10 targets, but by no more than 2–3 Å. Only 3 models out of all FR/A targets were worse than those selected as the first by the Kolinski-Bujnicki group (by about 0.6–1.5 Å) and only one model out of all NF targets (by 1.4 Å). The accuracy of the contact data for these 4 targets was either below 15% or above 30% and this confirmed our hypothesis formulated in the previous section that the most useful contact predictions are typically of 15–30% accuracy.

**Figure 3:**
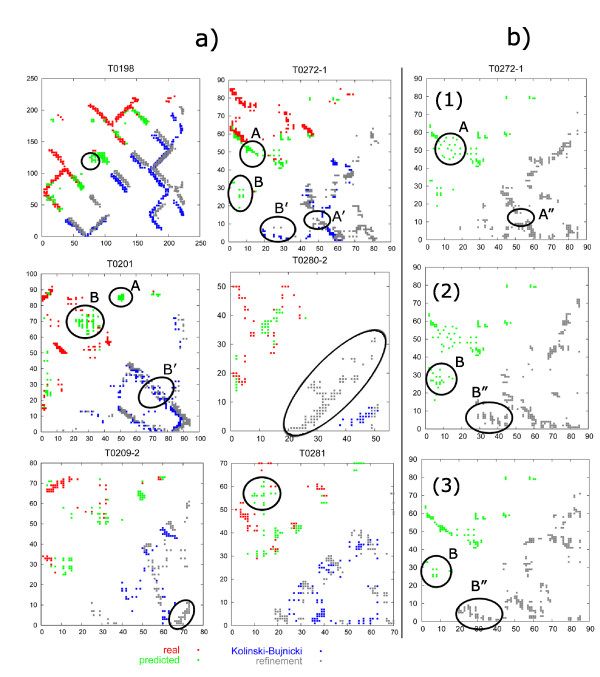
**Contact maps presenting results of fold-refinement simulations**. The contact maps of the selected CASP6 targets are presented in (a). In the upper triangle in each contact map real (red) and predicted (green) contacts are compared. In the bottom triangle a contact map of Kolinski-Bujnicki's first model (blue) is superposed on a contact map of the final model obtained after the refinement simulations (grey). In most cases we observed improvement of the contact maps for models after the refinement. Some accurate contacts were rebuilt by the CABS despite not being preliminarily predicted (T0209-2). Some falsely predicted contacts in diffused clusters were not observed in the final model (T0281). Predicted contacts in dense and numerous clusters were observed almost in all cases (A and A' in the T0272-1 contact map), contrary to diffuse sparse contact clusters. (b) Lower triangles, contact maps of the T0272-1 models obtained after the simulations with restraints based on the data sets (upper triangles) with either the A or B group of contacts modified. (1) Reduction of the influence of restraints based on the A group of contacts on the simulation with respect to the original contact data in (a) by diffusing these contacts. Intensification of the effect of B contact-based restraints by increasing the number of these contacts (2) and by increasing the scaling factor in the restraint potential corresponding to these contacts(3).

In Figure 3, we compared the real contact maps with predicted contact maps of selected targets. We also compared the contact maps generated from models before and after the refinement simulations. It is worth noticing that the contact map of the final refined protein model does not necessarily overlap entirely with the predicted contact map used for generating restraints for simulations. It is the consequence of the way the restraint potential is constructed (see **Methods **section), i.e. to allow a fraction (typically 20–30%) of restraints to be ignored during refinement simulations. Such a form of the restraint potential is especially useful when only a small fraction of predicted contacts is accurate.

In most cases we observed that contact maps of the refined models were more accurate than those of Kolinski-Bujnicki's original models. We also noticed that some contacts which were visible in the contact map of the refined model were not provided by contact predictors (see selected contacts in the T0209-2 map in Figure 3a). This is the consequence of the interplay between the CABS force-field and the contact-based restraints in which CABS plays the key role. For example, the CABS tool is able to reach a protein energy minimum by rebuilding the conformation of one part of the protein chain to satisfy interactions defined either by its force field or by contact-based restraints, imposed on the other, even distant part of the protein (see T0280-2 in Figure 3a).

Most of the contacts in the Kolinski-Bujnicki model of the T0198 target were uniformly shifted by a few residues with respect to the real contact map (see Figure 3a). Thanks to rather precisely predicted contact-based restraints the refinement simulation managed to shift these contacts back to the native-like pattern. However, the Cα RMSD of the final model did not improve significantly with respect to Kolinski-Bujnicki's results (from 9.8 to 8.1 Å). As it was tested by Ortiz et al.[28], if all contacts are slightly but uniformly shifted with respect to the native ones, the RMSD of the overall protein model is affected only to a minor extent (1–2 Å). Our results for the T0198 target confirm this observation.

Most of the predicted contacts for all targets are precise if they are accurate. The main problem with this kind of data is not the shift of the contact data but the existence of completely mispredicted contacts, very distant from the native ones in the contact map (see the A group of contacts in the T0201 map and A and B in the T0272-1 map in Figure 3a). Such mispredicted contacts, if satisfied in the simulations, could severely worsen the quality of the final model[4]. Of course, if such contacts disagree completely with the CABS-only generated energy landscape or satisfying them would require serious rebuilding of a conformation, not possible in the low-temperature refinement, they may not be observed in the final contact map (e.g. in the case of the A group in the T0201 contact map in Figure 3a). In other cases, their influence could be destructive (see T0280-2 in Figure 3a).

The effect of such false contacts could be reduced, as we observed on the basis of contact maps presented in Figure 3, by diffusing the uncertain contact data or decreasing their number. If some predicted contacts are concentrated and, what is more important, numerous in one area of the contact map, the component of the restraint potential corresponding to these contacts is high and significantly influences the folding process. Consequently, concentrated contacts are likely to be observed in the final models (see the dense groups of contacts in T0198, T0209-2 and T0280-2 contact maps in Figure 3a). Of course, this could be useful if contacts are accurate (the T0198 case). However, if they are suspected to be false, like in the case of the T0280-2, they should not be incorporated in the simulations in the form of populated and dense data set. On the other hand, if contacts in a certain area of the predicted contact map are diffuse, they are less numerous and, therefore, they modify the energy landscape to a lesser extent. More diffuse and less numerous contacts could even remain undetected in the final model (see selected contacts in the T0281 contact map in Figure 3a).

To confirm this observation, we tested several methods for reducing the influence of false contacts on structure modeling (see Figure 3b). We selected the T0272-1 case, for which two distinct false clusters of contacts were predicted. The A group of contacts forms a dense and numerous cluster and the contacts in the B group are less numerous and less close to each other (see Figure 3a). After the refinement simulation in the final model we observed mainly contacts from the A group (depicted as A'). The B group was barely visible (depicted as B'). To reduce the A group of contacts, the scaling factor of the potential component computed for this group of contacts could be decreased, the number of these contacts could be reduced and finally they could be more diffused (see A" in Figure 3b-1). To obtain the B contacts in the final protein model, on the other hand, the scaling factor should be increased (see B" in Figure 3b-3), the number of these contacts should be larger (see B" in Figure 3b-2) or they should form a more dense cluster. Our results of additional simulations for T0272-1 (see Figure 3b) confirmed that if a cluster of predicted contacts is more numerous and dense, it is more probable that we observe it in a final refined model. Decreasing the scaling factor of the restraint potential affects the simulation, but to a minor extent only.

Another important issue which we encountered is the frequent inconsistency of contacts obtained by the combination of different methods. For example, (see Figure 3a), a large group of contacts in the T0201 contact map marked as B, was obtained by combining contacts predicted by two methods. In the refined model this group of contacts was reduced to those contacts which were consistent (B'). As it was mentioned above, the specific form of the restraint potential enables us to suppress the effect of some inconsistent contacts, provided that the scaling factor is not too high. In such a way, accurate contacts could be distinguished from the false ones due to some geometry or physical or knowledge-based criteria defined in the CABS force field. This ability of the CABS algorithm could be especially useful when a diverse set of data from different sources is used.

The overall accuracy of predicted contacts may not be crucial in refinement simulations. For example in the case of T0281, T0230 and T0212 targets, for which the accuracy of contact maps was nearly 40%, the quality of the final models did not improve significantly after the simulations. This is a consequence of the fact that the most of the accurate contacts were already observed in the Kolinski-Bujnicki starting model and were provided either by CABS or Frankenstein-3D. The remaining contacts, though diffuse, were mispredicted. The contacts which were crucial for correct structure determination but were not observed in the starting models were also absent in the predicted sets (see the T0281 contact map in Figure 3a). Consequently, mispredicted contacts, despite being sparse, dominated the simulation driving the system into false minima.

According to our analysis of Figure 3, it appears that despite their frequent inconsistency more concentrated contact clusters with some accurate and some shifted contacts, but by a few residues only, are more useful for the refinement simulations with the CABS model than a diffuse set of precise contacts of higher overall accuracy.

### De novo folding supported by predicted contacts

If we compare de novo folding supported by predicted contacts with refinement simulations and the contact-based ranking (see Table 3), it turns out that the performance of this approach is in between these two above discussed methods. The results of the contact-based ranking are better in the case of contact sets of high coverage (1.5 N contacts or consensus from data for all predictors). On the contrary, the results of the refinement simulations are best for the data set of moderate, and thus well balanced, coverage and accuracy. Namely, the results for sets of N contacts are better than for 1.5 N contacts. Also, the best results of the refinement simulations are for the consensus from data from the best five predictors, for which the accuracy is better than in the case of consensus from three predictors, and the fraction of false contacts is smaller than for the consensus data from all predictors (see Table 2). It seems that during the REMC simulations the accuracy of contact data is more important than the coverage, because a large fraction of false contacts may disturb the protein folding pathway, and thus a near-native structure cannot be achieved. Regardless of which modeling method we chose, contact data obtained by the consensus of contact predictions performed better than simple combining together the top-scoring contacts of each prediction (compare results for N/2 top-scoring contacts from each of the best three predictors and for the consensus of the whole data from the best three predictors in Table 3).

**Figure 4:**
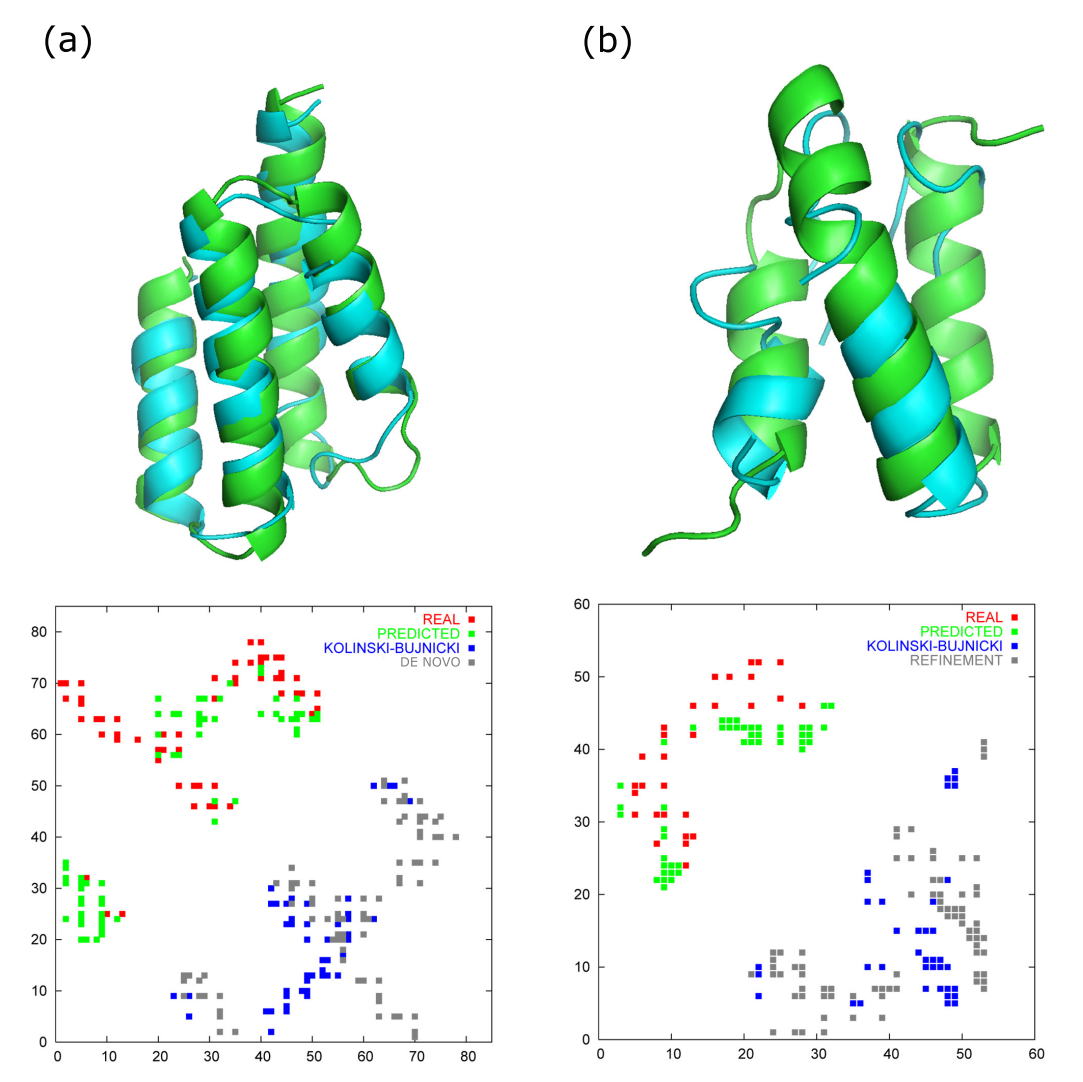
**Distinctive results of two simulation methods involving the predicted contacts: de novo folding and refinement**. Results of de novo folding (a) are represented by the best model of the T0248-1 target superimposed on the native structure (RMSD = 2.3 Å) and by the contact map with depicted real and quite accurate and precisely predicted contacts (upper triangle) and contacts of the best model obtained in the folding simulation and the first model of the Kolinski-Bujnicki group (lower triangle). Significant improvement of model quality and its contact map with respect to the native is observed. Results of the refinement simulations (b) are represented by the best model of the T0215 target (green) superimposed on the native structure (blue) (RMSD = 5.5 Å) and by the contact map constructed in the same fashion as in (a). Despite the low quality of the contact data predicted for the T0215 target the quality of the final refined model improved (but not significantly) in comparison to the original Kolinski-Bujnicki results (RMSD = 7.9 Å from the crystallographic structure Cα-trace).

In the case of T0198, mispredicted contacts (see selected contacts in the T0198 contact map in Figure 3a) were observed in the final model of de novo folding simulation (data not shown), but not in the model obtained in the refinement simulation. It is the consequence of the fact that refinement simulations enable us to neglect some mispredicted contacts if they diverge significantly from the starting model and require too radical rebuilding of the whole conformation, which is impossible due to too low temperature and too loose restraints. In de novo folding simulations, in which the starting temperature is significantly higher than the folding transition temperature, all contacts, both mispredicted and accurate, can be equally satisfied because the starting conformation can be freely rebuilt. Consequently, if the predicted contacts are not accurate enough, de novo folding simulation can lead to the completely false fold, especially when contact-based restraints are tight and thus satisfied in majority. On the other hand, if the prediction of contacts is very accurate and substantial, such de novo simulations exploit it better than refinement simulations, because the energy landscape is properly modified by these contacts nearly from the beginning of the folding process. In Figure 4 we present two distinct situations as an example of the most favourable approach to protein modeling: refinement simulations with low-quality contact data (target T0215) and de novo folding with accurate contact data (T0248-1).

The accuracy of contact prediction for T0248-1 is 25.8% and it covers all the important regions of the real contact map (mainly contacts between helices) with a tolerance of a few residues. Such high quality data are better exploited when used as restraints in de novo folding (the RMSD of the representative structure of the best cluster is 2.3 Å) rather than in refinement simulations (RMSD = 3.0 Å). For comparison, the best result of the Kolinski-Bujnicki group was 7.9 Å from the native structure (this model was submitted as the second). The accuracy of contact data for T0215 is only 11.8% and the restraints based on such contact data, if fully satisfied, could worsen the prediction. The refinement simulation, however, takes advantage of part of the restraints only rejecting those inconsistent with the starting model and improves the RMSD from 6.2 Å to 5.5 Å (the best model). In the case of de novo folding the best model obtained was 6.8 Å from the native structure.

In the case of poorly predicted contacts, e.g. for T0215, the quality of the final model does not deteriorate in the refinement as much as in the case of restrained de novo folding. As the average accuracy of contact predictions is still rather low, the refinement-based approach to protein structure modeling seems to be more useful.

## Conclusion

In this work we explored various methods for improving template-free modeling by using contact prediction. In the straightforward contact-based ranking of protein models, the best way is to combine as many predicted contacts as can be collected from different sources into a consensus set of high coverage and at least medium accuracy. Such combination of data obtained by different methods leads to a significant reduction of the effects of limitations and errors of each method.

Introducing contact-based restraints into the template-free REMC simulations requires high accuracy or at least a significant number of accurate and semi-accurate contacts and only few false ones. Covering the most of the real contact map by predicted contacts improves structure prediction but overall, coverage is of lesser importance than accuracy. Contacts which are predicted with low probability and therefore can be false should be more diffuse and less numerous in the data set to reduce their influence on the conformational energy. The CABS force field is able to suppress the effect of the wrongly predicted contacts during the REMC simulations, provided that they do not form a densely-populated clusters in the predicted contact map.

In general, we have shown that the theoretically predicted contacts could be useful in protein modeling, providing some other high-performance modeling tool, such as CABS, is also used. The usage of additional modeling tool is inevitable due to rather low accuracy of contact data, insufficient for the direct reconstruction of the 3D model. Predicted contacts can be used in simple and straightforward model ranking, refinement of crude models and de novo folding simulations. On average, all approaches lead to the improvement of the quality of predicted models. Sometimes the improvements are of a qualitative nature. This study provides a guideline how to use the contact prediction methods at various stages of protein structure prediction.

The method described here is not restricted to the use of data from contact predictors. It is possible to employ contacts provided by any other method, theoretical or experimental. They can be extracted from very rough or coarse structural alignments or from fuzzy experimental data, for example from ambiguous NOEs or cross-link data. The initial structures for the most successful refinement simulations we obtained can be provided by any available modeling method from fold-recognition to entirely de novo methods.

Although we tested various methods for contact-assisted model building, refining and ranking using only the CABS generated models, it seems to be rather safe to expect similar applicability of predicted contacts in other modeling techniques.

## Abbreviations

NF: New Fold; FR: Fold Recognition; FR/A: Fold Recognition – Analogy; FR/H: Fold Recognition – Homology; CM: Comparative Modeling; CASP6: the Sixth Critical Assessment of Techniques for Protein Structure Prediction; CABS: algorithm based on Cα, Cβ and the side group centre of an amino acid; REMC: Replica Exchange Monte Carlo method.

## Authors' contributions

DL prepared the contact data sets and performed the refinement and de novo folding simulations, the ranking of CASP6 models, statistical analysis, assembled figures and wrote the draft of the manuscript. AK provided CASP6 decoys generated by the Kolinski-Bujnicki group, conceived the study, and participated in its design. Both authors read and approved the final manuscript.
